# Screening for Health-Related Social Needs Among Inpatient Adults at an Academic Medical Center

**DOI:** 10.1007/s11606-025-09848-1

**Published:** 2025-10-16

**Authors:** Jocelyn Carter, Narmeen Rehman, Adaugo Amobi, Natalia Swack, Yu Otaki, Brammy Rajakumar, Sammer Marzouk

**Affiliations:** 1 Division of General Internal Medicine, Massachusetts General Hospital, Boston, MA 02114 USA; 2 Wayne State University School of Medicine, Detroit, MI 48201 USA; 3 Department of Medicine, Massachusetts General Hospital, Boston, MA 02114 USA; 4 Tufts University, Medford, MA 02115 USA; 5 Harvard Medical School, Boston, MA 02115 USA; 6 Northwestern University Feinberg School of Medicine, Medical Scientist Training Program, Chicago, IL 60611 USA

**Keywords:** health-related social needs, social determinants of health, social work consultation, readmissions, inpatient, hospitalizations, hospital discharge

## INTRODUCTION

As medical expenditures and chronic disease in the US healthcare system continue to rise, hospitals face growing pressure to effectively identify and address unmet health-related social needs (HRSN) in clinical settings. While medical care contributes to only 10–20% of preventable health outcomes, non-clinical factors like HRSN drive the remaining 80–90% of health outcomes and are tied to avoidable utilization of acute care pathways.^1,2^ A mandate for routine inpatient HRSN screening from the Centers for Medicare and Medicaid emerged in 2024; however, most screening efforts occur in the outpatient or emergency settings. Inpatient screening remains understudied with limited information on best practice regarding workflow integration and how clinical outcomes may be impacted.

We conducted a pilot study to identify HRSNs on an internal medicine unit—specifically whether HRSNs led to social work (SW) consults, were documented in discharge summaries, or were associated with increased incidence of 30-day readmissions and emergency department (ED) use. By investigating these measures, we hoped to fill a literature gap and provide insights on inpatient social needs screening.

## METHODS

We conducted an observational study of adult inpatients (≥ 18 years) admitted to a 28-bed internal medicine unit (August 2021–March 2022). A standardized HRSN screening tool^3^ was administered across domains including food/housing insecurity, utilities/medication payments, transportation, employment, education, and childcare. Results were entered in REDCap and shared with clinical teams during multidisciplinary rounds.

The primary outcome was the prevalence of ≥ 1 unmet HRSN. Secondary outcomes included inclusion of HRSN in discharge summaries, a SW consult within < 3 days of hospitalization, and 30-day clinical outcomes (readmission and ED use). Demographics, comorbidities, and insurance type were collected from the electronic medical record. Analyses included univariate, chi-square, and logistic regression using SPSS v39.1. The pilot was exempt from IRB review.

## RESULTS

Of 155 screened (mean age 62.9, 41% female), 62% reported ≥ 1 unmet HRSN (Table 1). Most common needs included transportation (22%), and food insecurity (19%). Among those with unmet HRSN, 67% (*n* = 55) received a SW referral; 27% (*n* = 22) had social needs documented in discharge summaries.

**Table 1:** Participant Characteristics and Reported Unmet Social Needs

Participant characteristics and reported unmet social needs	*N* = 155
Age, years, mean (SD)	62.9 (16.1)
Female sex, *N* (%)	63 (40.6)
Race and ethnicity, *N* (%)	
Asian, non-Hispanic	4 (2.6)
Black, non-Hispanic	18 (11.6)
Hispanic/Latino	7 (4.5)
White, non-Hispanic	124 (80.0)
Other	2 (1.3)
Primary insurance, *N* (%)	
Medicare	48 (31.0)
Medicaid/MassHealth	34 (21.9)
Commercial/private	71 (45.8)
Other	2 (1.3)
Highest level of education, *N* (%)	
≤ High school	63 (40.6)
Some college or more	88 (56.8)
Did not answer/unknown	4 (2.6)
Primary reason for admission, ***N*** (%)	
Infectious disease	29 (18.7)
Gastroenterology	29 (18.7)
Cardiac	16 (10.3)
Respiratory	10 (6.5)
Psychiatry	9 (5.8)
Fall	9 (5.8)
Neurology	7 (4.5)
Pain	6 (3.9)
Hematology	6 (3.9)
Altered mental status	5 (3.2)
Glycemic instability	5 (3.2)
Nephrology	5 (3.2)
Other (hemodynamics, other vascular issues, oncology, failure to thrive, rheumatology, metabolics, immunocompromised, other)	19 (12.3)
Pre-existing diagnoses, ***N*** (%)	
Cardiac	107 (69.0)
Hypertension	73 (47.1)
Arrhythmia	42 (27.1)
NSTEMI/CAD	30 (19.4)
Congestive heart failure	30 (19.4)
Psychiatry	67 (43.2)
Alcohol abuse	32 (20.1)
Anxiety	29 (18.7)
Depression	26 (16.8)
Heroin/drug abuse	17 (11.0)
Gastroenterology	63 (40.1)
GERD	21 (13.5)
Cirrhosis	17 (11.0)
Respiratory	45 (29.0)
Asthma	18 (11.6)
COPD	17 (11.0)
Diabetes	43 (27.7)
Nephrology	39 (25.2)
CKD	29 (18.7)
Neurology	30 (19.4)
Oncology	23 (14.8)
Medical needs, ***N*** (%)	
Transportation for clinical issues	34 (21.9)
Difficulty paying for medications	19 (12.3)
Social or basic needs, *N* (%)	
Worried about not having enough food	32 (20.6)
Worried about not having housing moving forward	31 (20.0)
Food insecurity	29 (18.7)
No housing	21 (13.5)
Having trouble paying heat/electric	24 (15.5)
Moved 2–3 times in last 12 months	20 (12.9)
Employment, ***N*** (%)	
Unemployed and looking for work	25 (16.1)

SW consults were associated with decreased rates of readmission (*χ*^2^ (2) = 11.39, *p* = 0.003). Patients with a SW consult had a 30-day readmission rate of 16% compared to 19% without. Patients with ≥ 1 HRSN had a higher 30-day readmission rate (19.5% vs. 12.9%; *χ*^2^(1) = 0.78, *p* = 0.38).

**Figure 1:**
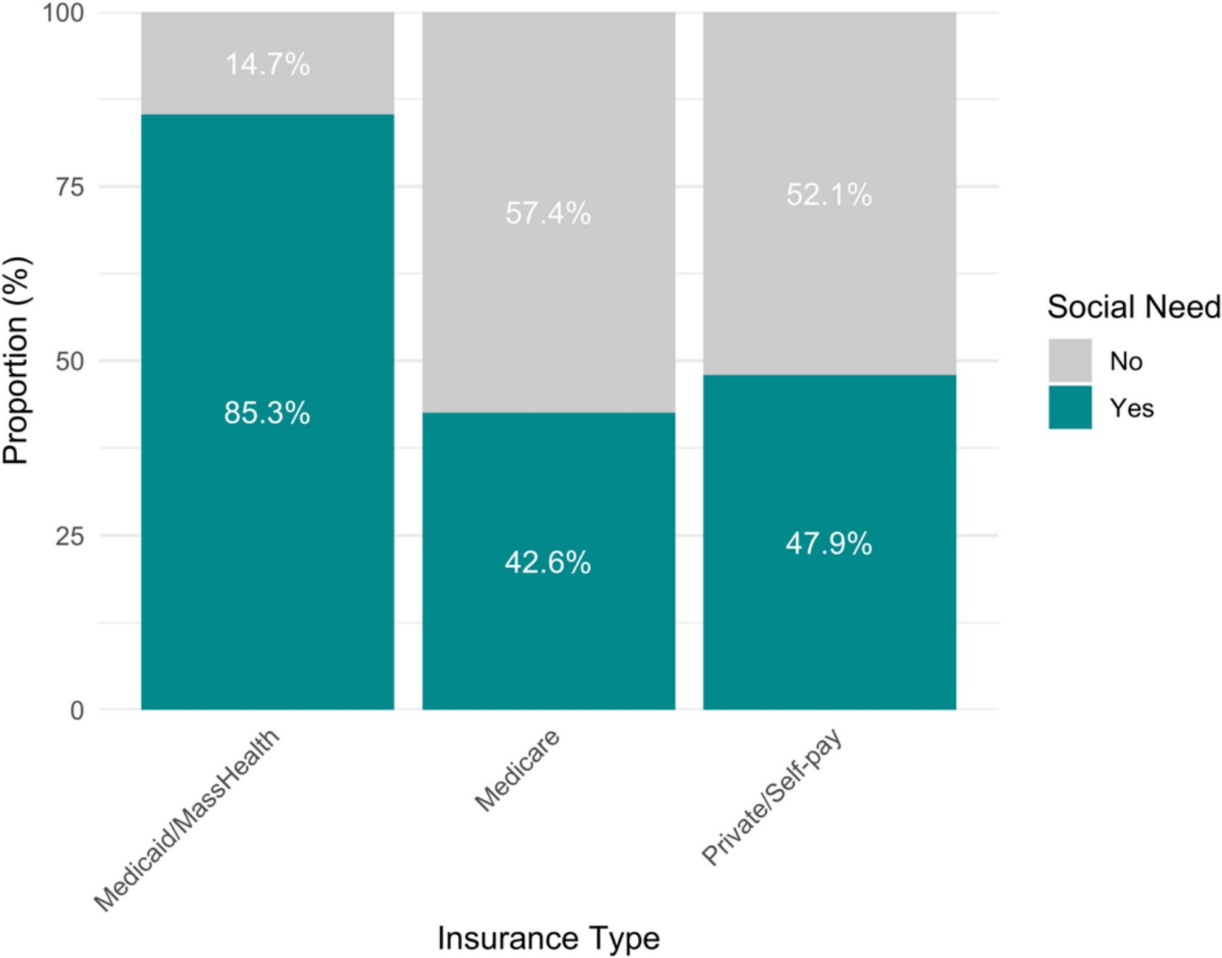
Proportion of individuals with unmet health-related social needs (HRSN) by insurance type.

Medicaid participants had the highest unmet need burden: 87% had ≥ 1 unmet need and 42% had ≥ 3 needs (Fig. 1). Insurance type was significantly associated with presence of HRSN (Fisher’s exact *p* < 0.001), with Medicaid participants being more likely to report food insecurity (44%) and employment instability (35%).

## DISCUSSION

This pilot highlights the feasibility and challenges of implementing inpatient HRSN screening. Over half (62%) of patients screened had ≥ 1 unmet HRSN, with transportation, food insecurity, and housing instability being the most frequently seen. Despite sharing screening results during multidisciplinary rounds, only half of patients with HRSNs had a SW consult and less than a third had unmet needs documented in discharge summaries. Barriers included limited pilot EHR integration, the lack of a standardized SW referral protocol, and inconsistent communication across rotating care teams. As expected, patients who received SW consults had lower 30-day readmission rates,^4^ while those with multiple HRSNs had higher readmission rates mirroring prior studies linking HRSNs to avoidable utilization.^5^

Our data also revealed Medicaid patients were disproportionately affected, with significantly higher rates of HRSNs and higher 30-day readmission rates.^5^ Prioritizing HRSNs screening (in connection with early care navigation) could better inform discharge planning for higher-risk populations.

Limitations included that only English-speaking participants on a single academic medical center unit were screened. Our institution’s resources for HRSN screening may not be directly comparable to those in other hospitals. Despite this, we believe that the barriers to capturing inpatient HRSNs seen here have important parallels in other settings and institutions.

These findings point to underexplored, yet actionable areas for inpatient HRSN screening. Strengthening inpatient screening will require solutions across internal (e.g., clinician training), organizational (e.g., data and referral infrastructure), and community (e.g., post-discharge coordination) domains.^6,7^ We plan to further explore these findings and potential solutions in a larger enterprise-wide population. Doing so will be critical to better assessment and standardization of inpatient social needs screening.

## Data Availability

De-identified data may be obtained upon request by contacting the corresponding author with a descriptive proposal stating the purpose of the data request.

## Declarations

### Disclaimer

The funder had no role in the design and conduct of the study; collection, management, analysis, and interpretation of the data; preparation, review, or approval of the manuscript; and decision to submit the manuscript for publication.

### Statement of Non-duplication

This manuscript is a unique submission and is not being considered for publication by any other source. This manuscript has not been published, in part or in full, in any form.
